# Supplementary material for: Comparative Analysis of Molecular Functions and Biological Role of Proteins from Cell-Free DNA-Protein Complexes Circulating in Plasma of Healthy Females and Breast Cancer Patients
Source: Int J Mol Sci. 2023 Apr 14;24(8):7279. doi: 10.3390/ijms24087279 (PMC10138639; doi:10.3390/ijms24087279)
Supplement: Supplementary file 1 [file ijms-24-07279-s001.zip › ijms-2320885-supplementary.pdf]

SUPPL TABLE S1 NPC proteins identified in the plasma of HF blood*

UniprotID	Protein Name	Gene Name	Score
P51665	26S proteasome non-ATPase regulatory subunit 7	PSMD7	66
Q92665	28S ribosomal protein S31, mitochondrial	MRPS31	70
Q9HD33	39S ribosomal protein L47, mitochondrial	MRPL47	56
Q6H8Q1	Actin-binding LIM protein 2	ABLIM2	61
Q8WXI4	Acyl-coenzyme A thioesterase 11	ACOT11	62
P40123	Adenylyl cyclase-associated protein 2	CAP2	66
P24298	Alanine aminotransferase 1	GPT	61
P18825	Alpha-2C adrenergic receptor	ADRA2C	57
Q7Z5R6	*Amyloid beta A4 precursor protein-binding family B member 1-interacting protein*	APBB1IP	63
Q96LR9	Apolipoprotein L domain-containing protein 1	APOLD1	62
O43918	Autoimmune regulator	AIRE	61
P08588	*Beta-1 adrenergic receptor*	ADRB1	60
Q6Y288	*Beta-1,3-glucosyltransferase*	B3GALTL	94
Q03060	*cAMP-responsive element modulator*	CREM	56
P08311	Cathepsin G	CTSG	68
P29973	cGMP-gated cation channel alpha-1	CNGA1	60
Q8TDX6	Chondroitin sulfate N-acetylgalactosaminyltransferase 1	CSGALNACT1	60
Q13111	Chromatin assembly factor 1 subunit A	CHAF1A	95
Q92187	CMP-N-acetylneuraminate-poly-alpha-2,8-sialyltransferase	ST8SIA4	78
Q96HJ3	Coiled-coil domain-containing protein 34	CCDC34	57
A6NFT4	Coiled-coil domain-containing protein 42B	CCDC42B	63
A2IDD5	*Coiled-coil domain-containing protein 78*	CCD78	70
Q86UT8	Coiled-coil domain-containing protein 84	CCDC84	58
P08174	Complement decay-accelerating factor	CD55	75
P36980	Complement factor H-related protein 2	CFHR2	72
Q9BR76	Coronin-1B	COR1B	93
P17812	CTP synthase 1	CTPS1	64
Q9P126	C-type lectin domain family 1 member B	CLEC1B	66
Q6NT55	Cytochrome P450 4F22	CYP4F22	59
Q7Z7J5	*Developmental pluripotency-associated protein 2*	DPPA2	71
P25205	DNA replication licensing factor MCM3	MCM3	61
O75190	DnaJ homolog subfamily B member 6	DNAJB6	96
O60941	Dystrobrevin beta	DTNB	65
Q8N7E2	E3 ubiquitin-protein ligase ZNF645	ZN645	58
O75354	Ectonucleoside triphosphate diphosphohydrolase 6	ENTPD6	61
A8MZ26	*EF-hand calcium-binding domain-containing protein 9*	EFCAB9	65
Q9BY07	*Electrogenic sodium bicarbonate cotransporter 4*	SLC4A5	78
Q6NXG1	Epithelial splicing regulatory protein 1	ESRP1	79
P55010	Eukaryotic translation initiation factor 5	EIF5	70

P14324	Farnesyl pyrophosphate synthase	FPPS	57
Q6PCT2	F-box/LRR-repeat protein 19	FBXL19	57
Q5T3I0	G patch domain-containing protein 4	GPATCH4	57
O96020	G1/S-specific cyclin-E2	CCNE2	82
Q99999	Galactosylceramide sulfotransferase	GAL3ST1	59
Q92990	Glomulin	GLMN	66
P06744	Glucose-6-phosphate isomerase	GPI	67
P23415	Glycine receptor subunit alpha-1	GLRA1	62
Q02108	*Guanylate cyclase soluble subunit alpha-3*	GUCY1A3	60
Q7LGA3	Heparan sulfate 2-O-sulfotransferase 1	HS2ST1	72
Q8WW32	High mobility group protein B4	HMGB4	60
O14929	Histone acetyltransferase type B catalytic subunit	HAT1	56
Q9BTM1	Histone H2A.J	H2AFJ	75
P31271	Homeobox protein Hox-A13	HOXA13	67
P17483	Homeobox protein Hox-B4	HOXB4	58
Q00444	*Homeobox protein Hox-C5*	HOXC5	92
P31273	Homeobox protein Hox-C8	HOXC8	65
Q92819	Hyaluronan synthase 2	HAS2	74
Q9NSI5	Immunoglobulin superfamily member 5	IGSF5	66
Q8NBZ0	*INO80 complex subunit E*	INO80E	89
P14735	*Insulin-degrading enzyme*	IDE	77
Q9NV88	Integrator complex subunit 9	INTS9	80
P20592	Interferon-induced GTP-binding protein Mx2	MX2	68
Q8IXL9	IQ domain-containing protein F2	IQCF2	56
O95198	Kelch-like protein 2	KLHL2	58
Q8N4N8	Kinesin-like protein KIF2B	KIF2B	60
O00522	*Krev interaction trapped protein 1*	KRIT1	70
Q13118	Krueppel-like factor 10	KLF10	68
Q03252	Lamin-B2	LMNB2	62
Q96BZ8	Leukocyte receptor cluster member 1	LENG1	68
P49137	MAP kinase-activated protein kinase 2	MAPKAPK2	86
A6NI15	Mesogenin-1	MSGN1	58
P02795	Metallothionein-2	MT2A	60
Q2M296	Methenyltetrahydrofolate synthase domain-containing protein	MTHFSD	57
Q96AQ8	*Mitochondrial calcium uniporter regulator 1*	CCDC90A	60
Q9BVV7	Mitochondrial import inner membrane translocase subunit Tim21	TIMM21	66
Q99558	*Mitogen-activated protein kinase kinase kinase 14*	MAP3K14	61
Q99683	Mitogen-activated protein kinase kinase kinase 5	MAP3K5	71
Q8NB16	Mixed lineage kinase domain-like protein	MLKL	68
P19105	*Myosin regulatory light chain 12A*	MYL12A	60
P24844	*Myosin regulatory light polypeptide 9*	MYL9	57
P48163	*NADP-dependent malic enzyme*	MAOX	73

Q9HD90	Neurogenic differentiation factor 4	NEUROD4	72
Q9Y639	Neuroplastin	NPTN	57
Q99784	Noelin	OLFM1	64
Q96PB7	Noelin-3	OLFM3	77
P48745	*NOV homolog*	NOV	57
Q8NGW1	Olfactory receptor 6B3	OR6B3	80
P30559	Oxytocin receptor	OXTR	73
Q15391	P2Y purinoceptor 14	P2RY14	56
Q9UQ90	*Paraplegin*	SPG7	61
Q9BRP8	Partner of Y14 and mago	WIBG	65
Q8IV76	PAS domain-containing protein 1	PASD1	66
O75570	*Peptide chain release factor 1, mitochondrial*	MTRF1	70
Q9NYL4	Peptidyl-prolyl cis-trans isomerase FKBP11	FKBP11	87
Q9BY49	Peroxisomal trans-2-enoyl-CoA reductase	PECR	63
Q9BUL5	PHD finger protein 23	PHF23	65
Q8N4E4	Phosducin-like protein 2	PDCL2	63
Q6NWY9	Pre-mRNA-processing factor 40 homolog B	PRPF40B	63
Q9H000	Probable E3 ubiquitin-protein ligase makorin-2	MKRN2	68
Q5JPH6	Probable glutamate--tRNA ligase, mitochondrial	EARS2	59
Q99680	*Probable G-protein coupled receptor 22*	GPR22	76
A2RTX5	Probable threonine-tRNA ligase 2, cytoplasmic	TARSL2	57
Q9ULL5	Proline-rich protein 12	PRR12	58
Q2TB18	Protein asteroid homolog 1	ASTE1	68
Q9UKY7	Protein CDV3 homolog	CDV3	61
Q13394	Protein mab-21-like 1	MAB21L1	57
O15151	Protein Mdm4	MDM4	58
P00734	Prothrombin	F2	59
Q5JUK9	*Putative G antigen family D member 1*	PAGE3	56
Q8N1L4	Putative inactive cytochrome P450 family member 4Z2	CYP4Z2P	59
Q5I0G3	Putative malate dehydrogenase 1B	MDH1B	86
Q9Y383	Putative RNA-binding protein Luc7-like 2	LUC7L2	61
Q5EBN2	Putative tripartite motif-containing protein 61	TRIM61	70
Q96NF6	Putative uncharacterized protein C8orf49	C8orf49	80
A8MUU9	Putative uncharacterized protein ENSP00000383309	YV023	66
A8MU76	*Putative UPF0607 protein ENSP00000381418*	N/A	60
A8MX80	*Putative UPF0607 protein ENSP00000383144*	YM017	68
Q9H974	Queuine tRNA-ribosyltransferase subunit QTRTD1	QTRTD1	62
Q3YEC7	Rab-like protein 6	RABL6	74
Q09MP3	RAD51-associated protein 2	RAD51AP2	62
Q86UC2	Radial spoke head protein 3 homolog	RSPH3	68
Q8IV61	Ras guanyl-releasing protein 3	RASGRP3	69
P20340	Ras-related protein Rab-6A	RAB6A	58

P11233	*Ras-related protein Ral-A*	RALA	59
P11234	Ras-related protein Ral-B	RALB	62
Q7Z6I6	Rho GTPase-activating protein 30	ARHGAP30	82
Q5TG30	Rho GTPase-activating protein 40	ARHGAP40	70
O43307	Rho guanine nucleotide exchange factor 9	ARHGEF9	57
P23443	Ribosomal protein S6 kinase beta-1	RPS6KB1	69
Q96CM3	RNA pseudouridylate synthase domain-containing protein 4	RPUSD4	58
Q9Y324	rRNA-processing protein FCF1 homolog	FCF1	64
Q2I0M5	R-spondin-4	RSPO4	67
Q92599	Septin-8	SEPT8	64
P34896	Serine hydroxymethyltransferase, cytosolic	SHMT1	78
Q13243	Serine/arginine-rich splicing factor 5	SRSF5	76
Q8WU08	*Serine/threonine-protein kinase 32A*	STK32A	68
Q86UX6	Serine/threonine-protein kinase 32C	STK32C	60
P50454	Serpin H1	SERPINH1	61
Q9BZQ2	SHC SH2 domain-binding protein 1-like protein	SHCBP1L	61
Q8IX30	Signal peptide, CUB and EGF-like domain-containing protein 3	SCUBE3	61
Q9NR83	SLC2A4 regulator	SLC2A4RG	75
P62314	*Small nuclear ribonucleoprotein Sm D1*	SNRPD1	65
Q9NYB5	Solute carrier organic anion transporter family member 1C1	SLCO1C1	80
Q86UG4	Solute carrier organic anion transporter family member 6A1	SLCO6A1	62
Q8NHX4	*Spermatogenesis-associated protein 3*	SPATA3	67
Q01081	*Splicing factor U2AF 35 kDa subunit*	U2AF1	112
Q8WXA9	Splicing regulatory glutamine/lysine-rich protein 1	SREK1	78
Q13033	*Striatin-3*	STRN3	66
Q7Z422	*SUZ domain-containing protein 1*	SZRD1	58
Q9BQG1	Synaptotagmin-3	SYT3	75
Q15573	TATA box-binding protein-associated factor RNA polymerase I subunit A	TAF1A	58
P17987	T-complex protein 1 subunit alpha	TCP1	81
Q9H2G4	*Testis-specific Y-encoded-like protein 2*	TSPYL2	79
Q13114	TNF receptor-associated factor 3	TRAF3	66
Q56UQ5	TPT1-like protein	TPT1L	68
P17535	*Transcription factor jun-D*	JUND	68
Q16514	Transcription initiation factor TFIID subunit 12	TAF12	68
Q00577	Transcriptional activator protein Pur-alpha	PURA	67
Q15629	*Translocating chain-associated membrane protein 1*	TRAM1	73
Q8N609	*Translocating chain-associated membrane protein 1-like 1*	TRAM1L1	62
Q99442	Translocation protein SEC62	SEC62	72
Q7Z5M5	Transmembrane channel-like protein 3	TMC3_	83
Q9NXH9	tRNA (guanine(26)-N(2))-dimethyltransferase	TRMT1	58
Q16560	U11/U12 small nuclear ribonucleoprotein 35 kDa protein	SNRNP35	65
Q15695	U2 small nuclear ribonucleoprotein auxiliary factor 35 kDa subunit-related protein 1	ZRSR1	80

C9J2P7	Ubiquitin carboxyl-terminal hydrolase 17-like protein 15	USP17L15	63
Q9UPT9	*Ubiquitin carboxyl-terminal hydrolase 22*	UBP22	68
Q16763	*Ubiquitin-conjugating enzyme E2*	UBE2S	63
Q9Y3C8	Ubiquitin-fold modifier-conjugating enzyme 1	UFC1	68
O75310	UDP-glucuronosyltransferase 2B11	UGT2B11	56
Q8IXR9	Uncharacterized protein C12orf56	C12orf56	56
Q8NEA5	Uncharacterized protein C19orf18	C19orf18	59
Q5T8R8	Uncharacterized protein C9orf66	C9orf66	60
Q5VIR6	Vacuolar protein sorting-associated protein 53 homolog	VPS53	62
O95670	V-type proton ATPase subunit G	ATP6V1G2	58
Q96JC4	Zinc finger protein 479	ZNF479	59

* Universal proteins are marked in italics.

SUPPL TABLE S2 NPC proteins identified in the plasma of BCP blood*

UniprotID	Protein Name	Gene Name	Score
Q9NZE8	39S ribosomal protein L35, mitochondrial	MRPL35	70
P32754	4-hydroxyphenylpyruvate dioxygenase	HPD	58
P46777	60S ribosomal protein L5	RPL5	60
P23526	Adenosylhomocysteinase	AHCY	58
Q969X2	Alpha-N-acetylgalactosaminide alpha-2,6-sialyltransferase 6	ST6GALNAC6	62
Q7Z5R6	*Amyloid beta A4 precursor protein-binding family B member 1-interacting protein*	APBB1IP	83
P48751	Anion exchange protein 3	SLC4A3	81
Q75V66	Anoctamin-5	ANO5	77
O95236	Apolipoprotein L3	APOL3	56
Q86W34	Archaemetzincin-2	AMZ2	59
Q8TF01	Arginine/serine-rich protein PNISR	PNISR	68
O95260	Arginyl-tRNA--protein transferase 1	ATE1	64
O15392	Baculoviral IAP repeat-containing protein 5	BIRC5	56
P08588	*Beta-1 adrenergic receptor*	ADRB1	67
Q6Y288	*Beta-1,3-glucosyltransferase*	B3GALTL	61
Q03060	*cAMP-responsive element modulator*	CREM	82
Q9NS84	Carbohydrate sulfotransferase 7	CHST7	70
Q9HCP0	Casein kinase I isoform gamma-1	CSNK1G1	93
Q5EG05	Caspase recruitment domain-containing protein 16	CARD16	204
P29466	Caspase-1	CASP1	64
Q8NEC5	Cation channel sperm-associated protein 1	CATSPER1	61
Q9H6E4	Coiled-coil domain-containing protein 134	CCDC134	57
A2IDD5	*Coiled-coil domain-containing protein 78*	CCDC78	58
Q03591	Complement factor H-related protein 1	CFHR1	60
P61201	COP9 signalosome complex subunit 2	COPS2	76
P21728	D(1A) dopamine receptor	DRD1	63
Q7Z7J5	*Developmental pluripotency-associated protein 2*	DPPA2	73

O75912	Diacylglycerol kinase iota	DGKI	60
O95886	Disks large-associated protein 3	DLGAP3	57
Q9H1X3	DnaJ homolog subfamily C member 25	DNAJC25	117
Q5T447	E3 ubiquitin-protein ligase HECTD3	HECTD3	63
Q9H6Y7	E3 ubiquitin-protein ligase RNF167	RNF167	62
Q6AZZ1	E3 ubiquitin-protein ligase TRIM68	TRIM68	68
Q05215	Early growth response protein 4	EGR4	67
Q16206	Ecto-NOX disulfide-thiol exchanger 2	ENOX2	58
A8MZ26	*EF-hand calcium-binding domain-containing protein 9*	EFCAB9	79
Q9BY07	*Electrogenic sodium bicarbonate cotransporter 4*	SLC4A5	62
A0FGR8	Extended synaptotagmin-2	ESYT2	60
Q14332	Frizzled-2	FZD2	72
O75084	Frizzled-7	FZD7	94
Q86XJ1	GAS2-like protein 3	GAS2L3	82
P48167	Glycine receptor subunit beta	GLRB	85
Q08379	Golgin subfamily A member 2	GOLGA2	63
Q99578	GTP-binding protein Rit2	RIT2	64
O95837	Guanine nucleotide-binding protein subunit alpha-14	GNA14	91
Q02108	*Guanylate cyclase soluble subunit alpha-3*	GUCY1A3	67
O96004	Heart- and neural crest derivatives-expressed protein 1	HAND1	61
Q92598	Heat shock protein 105 kDa	HSPH1	69
P60608	HERV-F(c)2_7q36.2 provirus ancestral Env polyprotein	EFC2_HUMAN	58
Q99626	Homeobox protein CDX-2	CDX2	65
P17482	Homeobox protein Hox-B9	HOXB9	62
Q00444	*Homeobox protein Hox-C5*	HOXC5	59
A6NJT0	Homeobox protein unc-4 homolog	UNCX	58
Q8NBZ0	*INO80 complex subunit E*	INO80E	77
P14735	*Insulin-degrading enzyme*	IDE	87
P14316	Interferon regulatory factor 2	IRF2	57
Q8IYV9	Izumo sperm-egg fusion protein 1	IZUMO1	60
Q5VZ72	Izumo sperm-egg fusion protein 3	IZUMO3	56
Q7Z3Y9	Keratin, type I cytoskeletal 26	K1C26	57
Q96EK5	KIF1-binding protein	KIAA1279	75
O00522	*Krev interaction trapped protein 1*	KRIT1	61
Q9BYE3	Late cornified envelope protein 3D	LCE3D_HUMAN	59
Q9NZU5	LIM and cysteine-rich domains protein 1	LMCD1	66
P06858	Lipoprotein lipase	LPL	63
Q68DH5	LMBR1 domain-containing protein 2	LMBRD2	57
Q9H239	Matrix metalloproteinase-28	MMP28	74
A0JLT2	Mediator of RNA polymerase II transcription subunit 19	MED19	70
Q96AQ8	*Mitochondrial calcium uniporter regulator 1*	CCDC90A	56
Q9P0P8	Mitochondrial transcription rescue factor 1	MTRES1	105

Q99558	*Mitogen-activated protein kinase kinase kinase 14*	MAP3K14	70
Q9P2K5	Myelin expression factor 2	MYEF2	64
P05976	Myosin light chain 1/3, skeletal muscle isoform	MYL1	60
P19105	*Myosin regulatory light chain 12A*	MYL12A	78
O14950	Myosin regulatory light chain 12B	MYL12B	57
P24844	*Myosin regulatory light polypeptide 9*	MYL9	72
P48163	*NADP-dependent malic enzyme*	ME1	59
E9PAV3	Nascent polypeptide-associated complex subunit alpha	NACA	57
Q99608	Necdin	NDN	78
O00401	Neural Wiskott-Aldrich syndrome protein	WASL	60
Q13562	Neurogenic differentiation factor 1	NEUROD1	66
P29371	Neuromedin-K receptor	TACR3	67
Q69YI7	Nuclear apoptosis-inducing factor 1	NAIF1	62
Q9Y3N9	Olfactory receptor 2W1	OR2W1	58
Q15645	Pachytene checkpoint protein 2 homolog	TRIP13	93
Q8TE04	Pantothenate kinase 1	PANK1	69
Q9UQ90	*Paraplegin*	SPG7	64
Q96NR3	Patched domain-containing protein 1	PTCHD1	65
O75570	*Peptide chain release factor 1, mitochondrial*	MTRF1	91
F5H284	Peptidyl-prolyl cis-trans isomerase A-like 4D	PPIAL4D	60
P23942	Peripherin-2	PRPH2	69
O43189	PHD finger protein 1	PHF1	67
Q9P215	Pogo transposable element with KRAB domain	POGK	69
Q9NZM6	Polycystic kidney disease 2-like 2 protein	PKD2L2	69
Q5SY16	Polynucleotide 5'-hydroxyl-kinase NOL9	NOL9	59
Q96KK3	Potassium voltage-gated channel subfamily S member 1	KCNS1	66
Q6PIU1	Potassium voltage-gated channel subfamily V member 1	KCNV1	60
P61758	Prefoldin subunit 3	VBP1	90
Q99680	*Probable G-protein coupled receptor 22*	GPR22	77
Q5T4B2	Probable inactive glycosyltransferase 25 family member 3	CERCAM	96
O95456	Proteasome assembly chaperone 1	PSMG1	66
O15234	Protein CASC3	CASC3	60
P48745	*Protein NOV homolog*	NOV	56
P49757	Protein numb homolog	NUMB	66
B4DS77	Protein shisa-9	SHISA9	68
P60059	Protein transport protein Sec61 subunit gamma	SEC61G	61
Q5JUK9	*Putative G antigen family D member 1*	PAGE3	61
Q06416	Putative POU domain, class 5, transcription factor 1B	POU5F1B	90
Q96IC2	Putative RNA exonuclease NEF-sp	44M2.3	60
Q9Y6Q9	Putative uncharacterized protein ENSP00000380701	YQ045_HUMAN	61
A8MU76	*Putative UPF0607 protein ENSP00000381418*	N/A	60
A8MV72	Putative UPF0607 protein ENSP00000382826	N/A	82

A8MX80	*Putative UPF0607 protein ENSP00000383144*	N/A	72
Q92670	Putative zinc finger protein 75C	ZNF75CP	57
P43487	Ran-specific GTPase-activating protein	RANBP1	56
Q15404	Ras suppressor protein 1	RSU1	63
P11233	*Ras-related protein Ral-A*	RALA	58
Q5HYW3	Retrotransposon gag domain-containing protein 4	RGAG4	62
Q9UJK0	Ribosome biogenesis protein TSR3 homolog	TSR3	72
A6NCQ9	RING finger protein 222	RNF222	93
Q5JTH9	RRP12-like protein	RRP12	70
Q8WU08	*Serine/threonine-protein kinase 32A*	STK32A	82
P48995	Short transient receptor potential channel 1	TRPC1	62
Q8N7X8	SIGLEC family-like protein 1	SIGLECL1	65
P62314	*Small nuclear ribonucleoprotein Sm D1*	SNRPD1	72
Q9UKG4	Solute carrier family 13 member 4	SLC13A4	61
Q9BQ15	SOSS complex subunit B1	NABP2	56
Q9HB58	Sp110 nuclear body protein	SP110	65
Q8NHX4	*Spermatogenesis-associated protein 3*	SPATA3	66
Q8NB90	Spermatogenesis-associated protein 5	SPATA5	58
Q01081	*Splicing factor U2AF 35 kDa subunit*	U2AF1	90
Q9Y3M8	StAR-related lipid transfer protein 13	STARD13	57
Q13033	*Striatin-3*	STRN3	87
Q7Z422	*SUZ domain-containing protein 1*	SZRD1	59
Q6STE5	SWI/SNF-related matrix-associated actin-dependent regulator of chromatin subfamily D member 3	SMARCD3	66
Q6XYQ8	Synaptotagmin-10	SYT10	60
Q9BXF9	Tektin-3	TEKT3	117
Q9H2G4	*Testis-specific Y-encoded-like protein 2*	TSYL2_HUMAN	69
Q49AM3	Tetratricopeptide repeat protein 31	TTC31	88
Q6PGP7	Tetratricopeptide repeat protein 37	TTC37	62
Q92623	Tetratricopeptide repeat protein 9A	TTC9	61
Q9BT49	THAP domain-containing protein 7	THAP7	68
P05412	Transcription factor AP-1	JUN	73
P17535	*Transcription factor jun-D*	JUND	68
Q9Y5Q3	Transcription factor MafB	MAFB	61
Q15629	*Translocating chain-associated membrane protein 1*	TRAM1	57
Q8N609	*Translocating chain-associated membrane protein 1-like 1*	TRAM1L1	56
Q9UM00	Transmembrane and coiled-coil domain-containing protein 1	TMCO1	66
Q96AN5	Transmembrane protein 143	TMEM143	74
Q9H813	Transmembrane protein 206	TMEM206	60
Q9Y2B1	Transmembrane protein 5	TMEM5	62
Q7Z4G4	tRNA (guanine(10)-N2)-methyltransferase homolog	TRMT11	58
Q9UJT0	Tubulin epsilon chain	TUBE1	65

Q13454	Tumor suppressor candidate 3	TUSC3	67
O75317	Ubiquitin carboxyl-terminal hydrolase 12	USP12	68
Q9UPT9	*Ubiquitin carboxyl-terminal hydrolase 22*	UBP22	93
Q8WUN7	Ubiquitin domain-containing protein 2	UBTD	58
Q16763	*Ubiquitin-conjugating enzyme E2 S*	UBE2S	63
Q96C57	Uncharacterized protein C12orf43	C12orf43	68
Q6ZW13	Uncharacterized protein C16orf86	C16orf86	93
O00159	Unconventional myosin-Ic	MYO1C	65
Q9NRQ5	UPF0443 protein C11orf75	C11orf75	56
P15692	Vascular endothelial growth factor A	VEGFA	61
O60504	Vinexin	SORBS3	66
Q52LC2	V-type proton ATPase subunit S1-like protein	ATP6AP1L	60
Q8TAF7	Zinc finger protein 461	ZNF461	58
Q96N20	Zinc finger protein 75A	ZNF75A	68
P51815	Zinc finger protein 75D	ZNF75D	57
Q9UPG8	Zinc finger protein PLAGL2	PLAGL2	61

* Universal proteins are marked in italics.
